# Stereoselective Synthesis of Flavonoids: A Brief Overview

**DOI:** 10.3390/molecules28010426

**Published:** 2023-01-03

**Authors:** Ana Margarida Pereira, Honorina Cidade, Maria Elizabeth Tiritan

**Affiliations:** 1 Laboratory of Organic and Pharmaceutical Chemistry, Department of Chemical Sciences, Faculty of Pharmacy, University of Porto, Rua Jorge de Viterbo Ferreira 228, 4050-313 Porto, Portugal; 2 CIIMAR—Interdisciplinary Centre of Marine and Environmental Research, University of Porto, Terminal de Cruzeiros do Porto de Leixões, Avenida General Norton de Matos, s/n, 4450-208 Matosinhos, Portugal; 3 TOXRUN—Toxicology Research Unit, University Institute of Health Sciences, CESPU, CRL, Rua Central de Gandra 1317, 4585-116 Gandra, Portugal

**Keywords:** flavonoids, enantiomers, enantioselective synthesis, chiral

## Abstract

Stereoselective synthesis has been emerging as a resourceful tool because it enables the obtaining of compounds with biological interest and high enantiomeric purity. Flavonoids are natural products with several biological activities. Owing to their biological potential and aiming to achieve enantiomerically pure forms, several methodologies of stereoselective synthesis have been implemented. Those approaches encompass stereoselective chalcone epoxidation, Sharpless asymmetric dihydroxylation, Mitsunobu reaction, and the cycloaddition of 1,4-benzoquinone. Chiral auxiliaries, organo-, organometallic, and biocatalysis, as well as the chiral pool approach were also employed with the goal of obtaining chiral bioactive flavonoids with a high enantiomeric ratio. Additionally, the employment of the Diels–Alder reaction based on the stereodivergent reaction on a racemic mixture strategy or using catalyst complexes to synthesise pure enantiomers of flavonoids was reported. Furthermore, biomimetic pathways displayed another approach as illustrated by the asymmetric coupling of 2-hydroxychalcones driven by visible light. Recently, an asymmetric transfer hydrogen-dynamic kinetic resolution was also applied to synthesise (*R,R*)-*cis*-alcohols which, in turn, would be used as building blocks for the stereoselective synthesis of flavonoids.

## 1. Introduction

**Figure 1:**
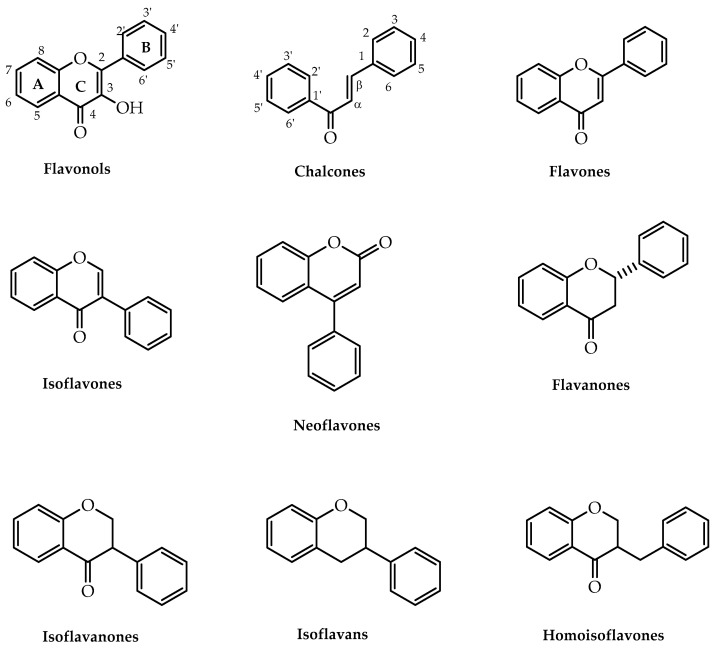
Main classes of flavonoids.

Flavonoids constitute a major group of polyphenolic compounds found in plants, fruits, vegetables, and nuts. They are associated with several roles in flora, namely, cell growth modulation and defence against extreme environmental conditions and oxidative stress. Moreover, they contribute to the perfume and colour in fruits and flowers, therefore promoting pollination [1,2]. In addition to flavonoids commonly found in terrestrial plants, some bioactive flavonoids can also be found in marine sources [3]. Structurally, flavonoids are composed of a 15-carbon scaffold with two aromatic rings (A and B) attached through a 3-carbon chain, which could be a heterocyclic ring denominated as a C ring. According to the degree of unsaturation and oxidation of the C ring and the position of the B ring, they can be categorised into different classes (Figure 1). In nature, this wide array of moieties is obtained through the combination of shikimate and acetate pathways under enzymatic transformation, with chalcones being the intermediates for the biosynthesis of the other classes of flavonoids [2,4].

Flavonoids are well-known to possess a variety of biological activities with therapeutic interests such as antioxidant [5], antimalarial [6], anti-inflammatory [7,8], antiviral [9,10], antibacterial [11], antidiabetic [8], antifungal [12], and anticancer [1,13,14,15,16] potential. It is also reported that they protect the cardiovascular system from oxidative stress as a consequence of their ROS scavenger ability [17]. Moreover, flavonoids can be employed in the cosmetic field as protective agents against skin deterioration and hyperpigmentation attributable to UV irradiation [18]. They also contribute to improving elasticity and skin strength as well as averting the occurrence of dark spots because of their inhibitory activity towards elastases, collagenases, and tyrosinases [18].

In addition to these diverse medicinal features, these polyphenolic compounds can be used in the food industry as sweeteners and colouring agents in pastry products [19]. Furthermore, they can function as flavour enhancers and protect against lipid peroxidation in seed oils and biscuits, owing to their antioxidant effect [19]. Flavonoids can also be employed in the textile area to produce biocompatible fibres and to ameliorate their quality [18]. Additionally, these natural compounds can be incorporated in the dyeing process of fibres for the purpose of procuring more environmentally friendly manufacturing [18]. It has also been reported that flavonoids possess the capacity to restrain metal corrosion, which arouses interest from a metallurgical field perspective [20].

Considering the biological and industrial potential of natural flavonoids, several chemical methodologies have been developed to obtain nature-inspired flavonoids, as summarised in Table 1 [21,22,23,24,25,26,27,28,29,30,31,32].

**Figure 2:**
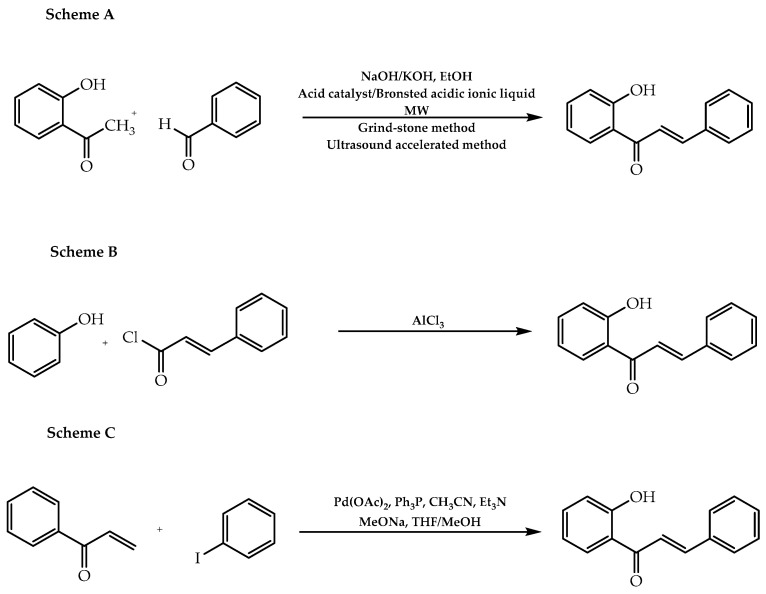
Synthetic methodologies of 2′-hidroxychalcones. **Scheme A:** Claisen–Schmidt reaction; **Scheme B:** Friedel–Crafts condensation; **Scheme C:** Heck coupling reaction.

As in nature, 2′-hydroxychalcones can be intermediates for the synthesis of other classes of flavonoids, such as flavonols, flavones, and flavanones. Synthetically, they can be obtained via Claisen–Schmidt [21], Friedel–Crafts, and Heck coupling pathways [21] (Figure 2). Regarding the Claisen–Schmidt reaction, it comprises the reaction of an aromatic aldehyde and a substituted acetophenone under basic catalysis (Scheme A, Figure 2). This process can be improved with recourse to microwave and ultrasound [30,33], resulting in the enhancement of the yields and a reduction in the reaction time [31]. With respect to the Friedel–Crafts method, 2′-hydroxychalcones are originated from the condensation of (*E*)-3-phenylprop-2-enoyl chloride and phenols through AlCl_3_ catalysis [21] (Scheme B, Figure 2). In addition, the Heck coupling pathway is based on the combination of aryl α,β-unsaturated ketone and iodobenzene, culminating in the formation of the desired chalcone [32] (Scheme C, Figure 2).

**Figure 3:**
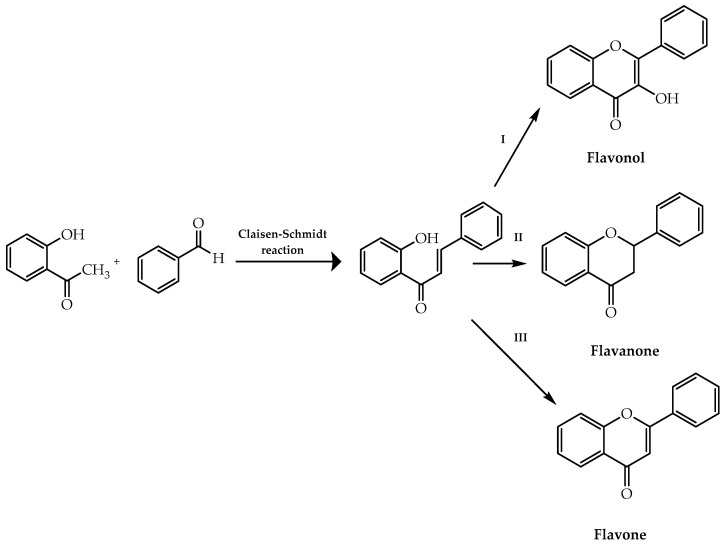
Synthesis of flavonols, flavanones, and flavones using 2′-hydroxychalcones as building blocks.

The Algar–Flynn–Oyamada methodology comprises the transformation of 2′-hydroxychalcones into flavonols (route I, Figure 3) through oxidative cyclisation mediated by hydrogen peroxide in alkaline medium [21,34,35]. The 2′-hydroxychalcones can also be building blocks for the synthesis of flavanones (route II, Figure 3) and flavones (route III, Figure 3). Considering the first class of flavonoids mentioned, they can be obtained through intramolecular cyclisation under acidic [36] or basic conditions [37], thermolysis [38], electrolysis [39], photolysis [40], microwave irradiation [41], a greener catalytic process [42], and palladium(II) catalysis [43]. Regarding flavones, these compounds can be synthesised through oxidative cyclisation under several reaction conditions such as classic I_2_-DMSO methodology [44] or using NH_4_I in a solvent-free environment [45]. There has also been reported the use of phenyliodinium acetate (PIDA) [46], selenium (IV) reagents under microwave irradiation [47], indium (III) halides in a gel-silica support system [48], CuI-mediated catalysis in the ionic liquid [bmim] [NTf_2_] as solvent [49], diphenyl disulfide at high temperatures [50], and oxalic acid-mediated catalysis [51] to obtain flavones via chalcones.

**Figure 4:**
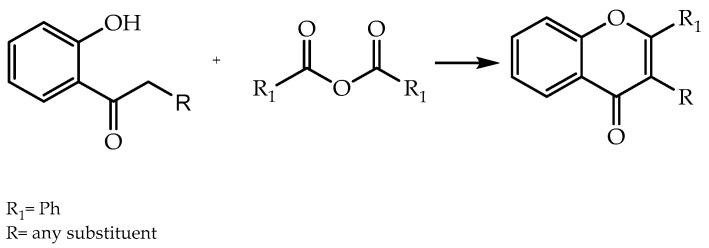
Synthesis of flavones and isoflavones by Allan–Robinson reaction.

Alternatively, flavones can be obtained by other methods. The Allan–Robinson approach is established as a synthetic route to produce flavones and isoflavones from the condensation of *o*-hydroxyaryl ketones, aromatic acid anhydride, and the sodium salt of correlated aryl carboxylic acid anhydride [21,52] (Figure 4) [53].

**Figure 5:**
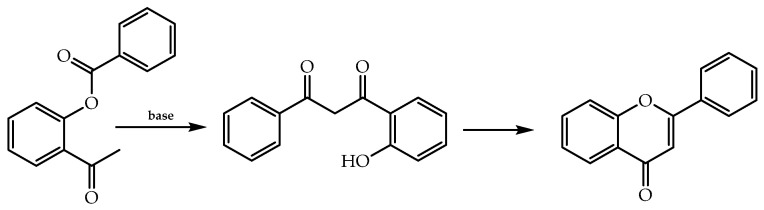
Synthesis of flavones by Baker–Venkataraman reaction.

The Baker–Venkataraman is another methodology implemented to produce intermediaries for the flavones’ synthesis [21], in which an α-acyloxy ketone is converted into β-diketones via basic catalysis and, subsequently, a cyclisation occurs to obtain the final flavone [54] (Figure 5).

**Figure 6:**
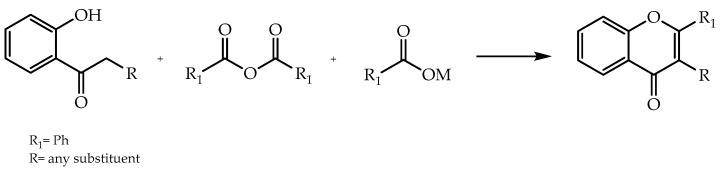
Synthesis of flavones by Kostanecki reaction.

The Kostanecki method is another well-known reaction pathway to obtain flavonoids, namely flavones. It consists of the combination between a *o*-hydroxyaryl ketone, aromatic acid anhydrides, and their related salt [55] (Figure 6). There are several reports of the application of this process to synthesise flavonoids with biological activity, namely the work developed by DeMeyer et al. [56].

**Figure 7:**
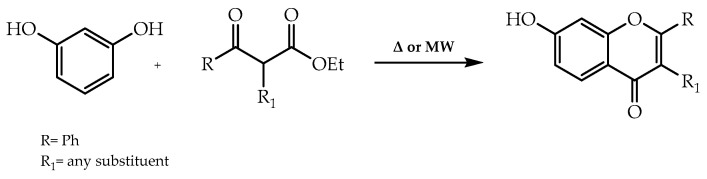
Synthesis of flavones by Mentzer reaction.

The Mentzer pyrone process encompasses the use of a phenol and a β-ketoester to synthesise flavone derivatives [57] without solvent and at high temperatures during a prolonged period of time or employing micro-wave irradiation [58] (Figure 7). A recent application of this strategy was employed by Pereira et al. [59] in the synthesis of flavones with antifouling activity [59].

**Figure 8:**
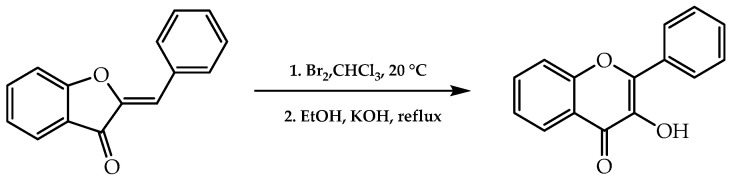
Synthesis of flavonols by Karl von Auwers approach.

The Karl von Auwers method comprises a set of reactions which transforms aurones into flavonols [60] (Figure 8). These molecules are essential in plants to ensure protection against UV irradiation and metallic ions due to their chelating feature and free radical scavengers. As a result, flavonols could be employed as a vehicle of treatment for pathologies associated with oxidative stress [61].

The Suzuki–Miyaura approach has been latterly implemented in flavonoid moieties synthesis [62]. It involves a cross-coupling reaction between an organohalide and boronic acid/esters in the presence of a palladium complex [63]. Its application is generally associated with the formation of chalcones, flavones, isoflavones, and neoflavones because palladium input occurs in an sp^2^-hybridised carbon–halide bond [62]. Hurtová et al. [64] applied this methodology to synthesise derivatives of quercetin, luteolin, chrysin, and flavonoid boronates.

More information about the progress in the synthesis of flavonoids is reported in a recent revision [65]. Despite the presence of the stereogenic centre in many scaffolds of flavonoids, most of the synthetic strategies ignore the stereochemistry of their structures.

**Table 1:** Summary of synthetic methods for flavonoid classes.

Flavonoids	Synthetic Approaches	References
Chalcones	Claisen–Schmidt reaction	[21,30,31]
	Friedel–Crafts reaction	[21]
	Heck coupling	[32]
	Suzuki–Miyaura reaction	[62]
Flavonols	Algar–Flynn–Oyamada reaction	[21,34]
	Karl von Auwers reaction	[60]
	Kostanecki methodology	[29]
Flavanones	Intramolecular cyclisation of 2′-hydroxychalcones	[36,37,38,39,40,41,42,43]
Flavones	Oxidative cyclisation of 2′-hydroxychalcones	[44,45,46,47,48,49,50,51]
	Allan–Robinson reaction	[21,52]
	Baker–Venkataraman reaction	[21,54]
	Kostanecki reaction	[55]
	Mentzer pyrone synthesis	[57]
	Suzuki–Miyaura reaction	[62]
Isoflavones	Allan–Robinson reaction	[21,52]
	Suzuki–Miyaura reaction	[62]
	Deoxybenzoin route	[22]
	Reductive cleavage of isoxazoles	[23]
	Intramolecular ketene cycloaddition followed by decarboxylation	
	Rearrangement and cyclisation of chalcone epoxides	[24]
	Rearrangement of flavanones	
	Wacker–Cook tandem conversion of α-methylene deoxybenzoins	[25]
	Cu(I)-mediated cyclisation of 3-(2-bromophenyl)-3-oxopropanol	[26]
Neoflavones	Suzuki–Miyaura reaction	[62]
	Pechmann reaction	[27]
	Perkin reaction	
	Wittig reaction of benzophenones	
	Metal-catalysed cross-coupling reactions such as Stille type	
	Direct arylation by the palladium-catalysed oxidative Heck coupling of arylboronic acids to coumarins	[28]

## 2. Stereoselective Synthesis of Flavonoids

Due to their biological activities and current concernment in attaining enantiomerically pure forms, chiral flavonoids are gaining attention in the scientific field [66]. The isolation of these natural compounds can be time-consuming and associated with a low yield, which accentuate even more the demand for the synthesis of enantiomerically pure forms of them [67].

A variety of methodologies to produce these bioactive compounds with high enantiomeric excess and purity have been reported. These approaches include separation processes, such as chiral chromatography [68,69,70,71,72], and stereoselective synthesis.

This revision complies with the research for synthetic routes of flavonoids with enantiomeric purity. For the purpose of supplementing this requirement, several methodologies have been developed.

### 2.1. Stereoselective Chalcone Epoxidation Approach

**Figure 9:**
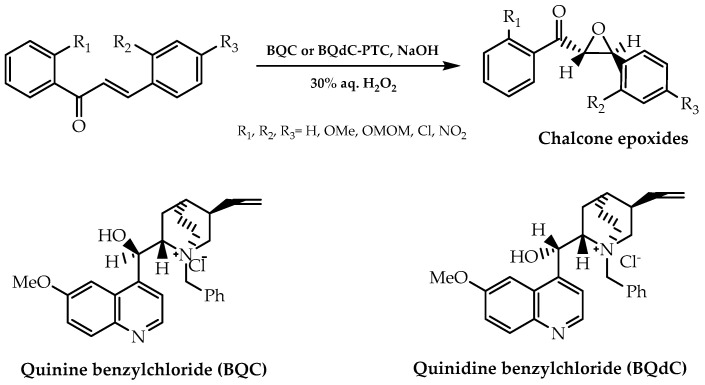
Chalcone epoxides synthesis using BQC and BQdC as phase-transfer catalysts.

As previously mentioned, chalcones play a major role as intermediaries for the synthesis of the various groups of flavonoids and, as a result, an asymmetric synthetic process was developed considering chalcones as building blocks. This procedure consisted of the asymmetric epoxidation of chalcones, giving rise to the respective epoxides and their later use as chirons for the synthesis of other flavonoids [73]. In 1976, the use of quinine benzylchloride and quinidine benzylchloride as chiral phase-transfer catalysts in the epoxidation of α,β-unsatured ketones was reported [74] (Figure 9), allowing the application of this method in the synthesis of chalcone epoxides. However, the resulting enantiomeric excess was low, therefore leading to investigations with the aim of improving enantioselectivity [74].

**Figure 10:**
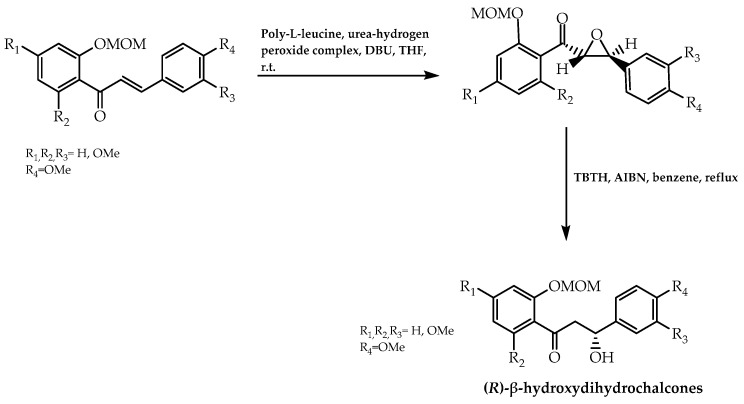
Hydroxydihydrochalcones synthesis via chalcone asymmetric epoxidation in a two-phase non-aqueous system and catalysed by poly-amino acids.

The turning point in this synthetic process arose from the implementation of three reaction components developed by Juliá et al. [75], comprising alkaline hydrogen peroxide, an organic solvent (carbon tetrachloride or toluene), and polymeric L- or D-alanine [75]. This synthetic process was later refined in a two-phase non-aqueous system in order to achieve higher enantiomeric purity [76]. Taking this into account, Nel et al. [77] proceeded to synthesise a series of enantiomeric (*S*)- and (*R*)-2′-methoxymethyl-β-hydroxydihydrochalcones (Figure 10), presenting some of them as an enantiomeric excess value in a range between 84% and 91%. These compounds constitute a resourceful tool in the industry, namely as sweeteners in candies and mouthwashes. Moreover, they assume a function of attracting insects in order to promote pollination in flora [77].

### 2.2. Sharpless Asymmetric Dihydroxylation, Mitsunobu Reaction, and Cycloaddition of 1,4-Benzoquinone

**Figure 11:**
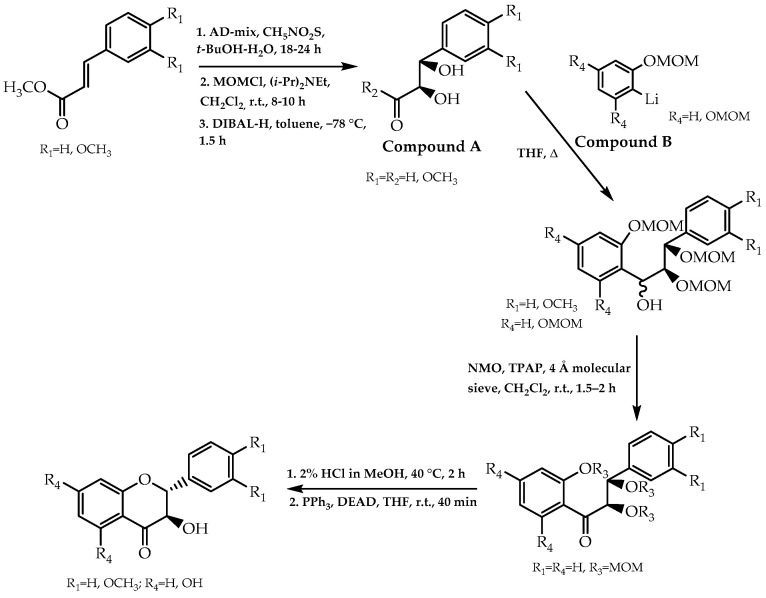
Stereoselective synthesis of 3-hydroxyflavanones based on the combination of Sharpless asymmetric dihydroxylation and Mitsunobu reaction.

In 2000, the combination of the Sharpless asymmetric dihydroxylation and Mitsunobu reaction was applied to obtain pure enantiomeric 3-hydroxyflavanones, resulting in a novel approach to synthesise this flavonoid class. The first reaction phase consisted of the formation of the (2*R*,3*S*)-diols (compound A, Figure 11) via Sharpless asymmetric dihydroxylation using AD-mix with an outstanding enantiomeric excess of 99%. The synthesis of the enantiomerically pure 3-hydroxyflavanones in the final phase was based on the intramolecular Mitsunobu pathway as verified in the configuration of the stereogenic centre (Figure 11). This methodology was also used to obtain (2*R*,3*R*)-3′,4′-*O*-dimethyltaxifolin, which is a derivative of a 3-hydroxyflavanone with a protective role in the hepatic system [78].

**Figure 12:**
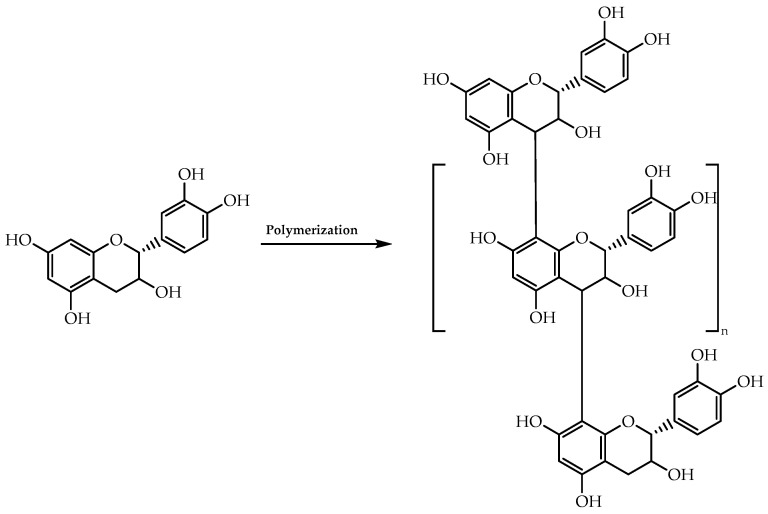
General structural feature of a natural condensed tannin composed of chiral flavan-3-ols.

The applicability of Sharpless asymmetric dihydroxylation extends to the synthesis of flavan-3-ols and isoflavonoid derivatives. Van Rensburg et al. [79] employed this methodology to synthesise polyoxygenated diarylpropan-1,2-diols from *retro*-chalcones, which would be then used to obtain the chiral flavan-3-ol scaffold [79]. These chiral moieties arouse interest in many fields, namely as building blocks of condensed tannins polymers (Figure 12), which have been receiving attention for the development of eco-friendly food packaging, owing to their chemical properties [80].

**Figure 13:**
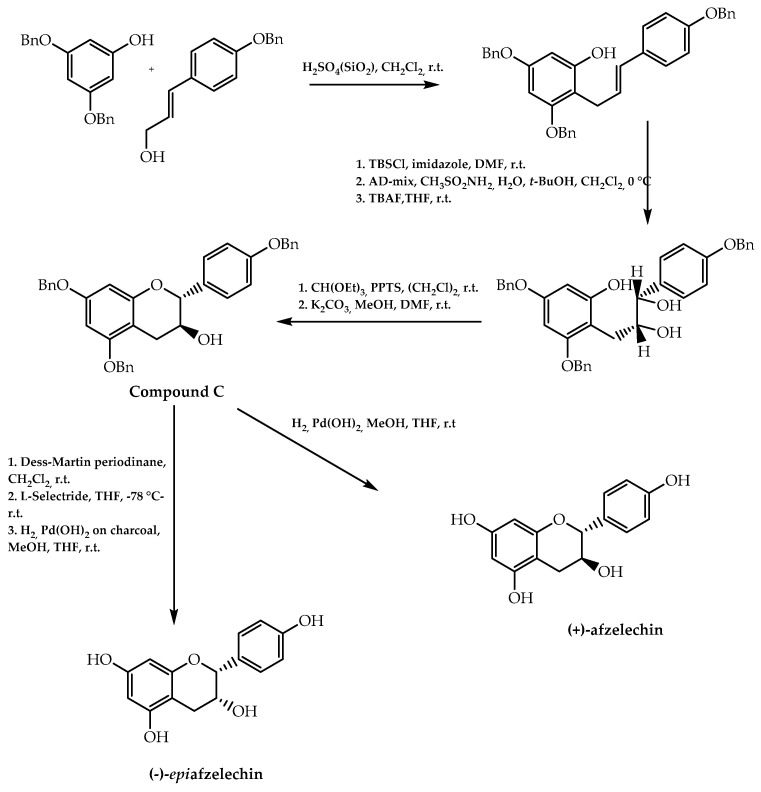
(+)-Afzelechin and (−)-*epi*afzelechin synthesis.

**Figure 14:**
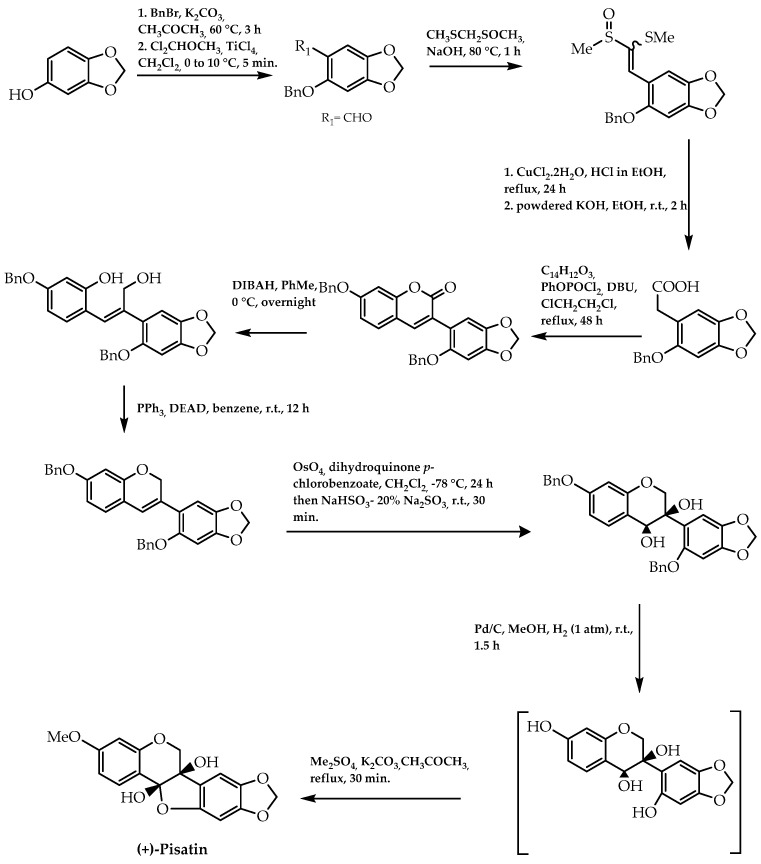
(+)-Pisatin synthesis.

This approach was also extended for the synthesis of (+)-afzelechin and (-)-*epi*afzelechin by Wan et al. [81] with the aim of obtaining analogues of *epi*gallocatechin-3-gallate with a cancer-preventive effect. These flavan-3-ols were stereoselectivity synthesised through the establishment of the stereogenic centres in the flavanol intermediate (compound C, Figure 13) by Sharpless dihydroxylation [81]. Moreover, (+)-pisatin, a natural isoflavonoid with a protective effect against microbial infections, was synthesised encompassing a Sharpless asymmetric dihydroxylation in one of the mechanism steps, resulting in an enantiomeric excess of 94% [82] (reaction phase 7, Figure 14).

**Figure 15:**
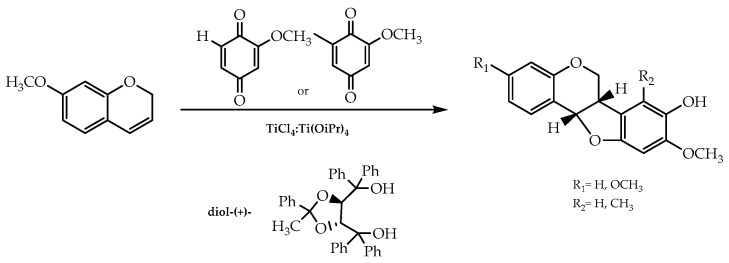
Pterocarpans obtained by cycloaddition via Ti-TADDOLate complex catalysis.

Furthermore, it was reported that isoflavonoid derivatives could also be obtained in an enantiomerically pure form via the cycloaddition of 1,4-benzoquinone and 2*H*-chromenes catalysed by a Ti-TADDOLate complex, which was demonstrated by Engler et al. [83]. They applied this procedure to synthesise pterocarpans with 75% and 80% of enantiomeric excess in light of their relevance as antifungal and antibacterial agents [83] (Figure 15).

### 2.3. Chiral Auxliaries Approach

**Figure 16:**
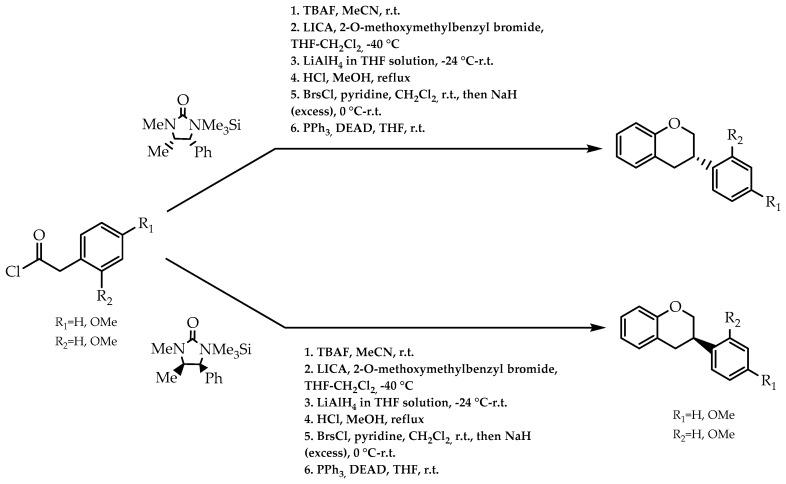
Enantioselective synthesis of isoflavans using imidazolidin-2-ones as chiral auxiliaries.

Isoflavans are a group of isoflavonoids with a variety of biological effects [84]. Since these compounds belong to a series of molecules where stereogenic centres are confined in 2, 3, and 4 positions, the development of enantioselective pathways to achieve enantiomeric pure moieties at position 3 could unfold stereoselective routes to other similar structures. Regarding this, Versteeg et al. [85] attempted to obtain isoflavans through a stereoselective α-benzylation of phenyl acetic acid derivatives, using (4*S*,5*R*)-(+)- and (4*R*,5*S*)-(-)-imidazolidin-2-ones as chiral auxiliaries (Figure 16). The implementation of this protocol brought an excellent outcome, with an array of enantiomeric excess between 94% and 99% and a chiral synthetic route for the 3-phenylchroman moiety [85].

**Figure 17:**
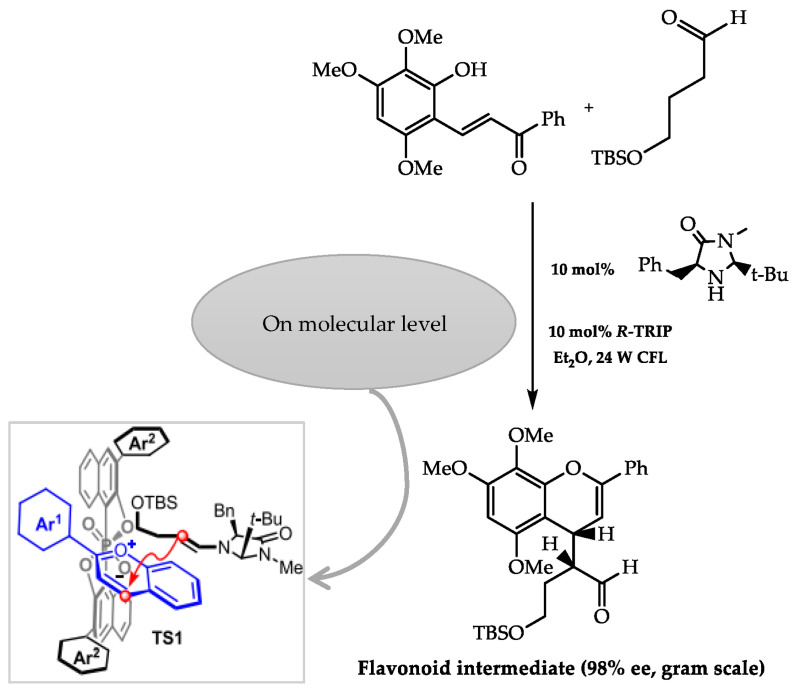
Stereoselective synthesis of flavonoid intermediate in ent-fissistigmatin-C synthesis (adapted from Xu et al.) [86].

In addition, imidazolidinones were also relevant as chiral auxiliaries in the total synthesis of *ent*-fissistigmatin C. This molecule structure embodies a fragment of a flavonoid and another of the sesquiterpenoid linked through carbon 4 and carbon 1″, which establishes the generation of two stereogenic centres in the natural compound. Xu et al. [86] developed a strategy to obtain fissistigmatin-C based on the reaction of a 2-hydroxychalcone and an aliphatic aldehyde [86]. In this reaction step, the flavonoid formed from the coupling of the two compounds previously mentioned was synthesised via a collaborative catalytic action of a chiral imidazolidine, (*R*)-TRIP, and visible light (Figure 17). On the molecular level, (*R*)-TRIP facilitated the attack of the enamine of the imidazolidinone in the *si* face by alleviating the steric hindrance, culminating with the formation of the flavonoid intermediate with 98% of the enantiomeric excess [86] (Figure 17).

**Figure 18:**
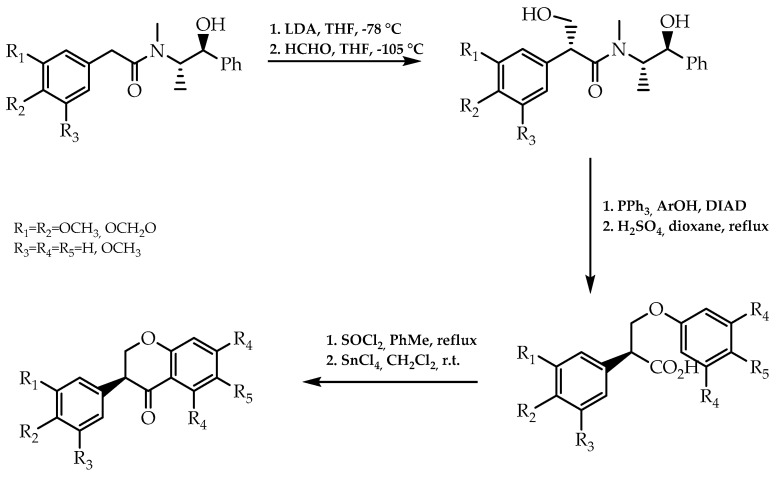
Enantioselective synthesis of isoflavanones using (*S,S*)-(+)-pseudoephedrine as chiral auxiliary.

Considering the bioactive potential of isoflavanones as antifungal and antibacterial agents, in 2000, an enantioselective synthesis of isoflavanones was reported by Vicario et al. [87]. They resorted an asymmetric aldol reaction between (*S*,*S*)-(+)-pseudoephedrine arylacetamides and formaldehyde to introduce chirality in the intended compound. Subsequently, it was given the synthesis of the B ring via aryl ether formation and the displacement of the chiral auxiliary, culminating in the formation of the desired isoflavanones through Friedel, Crafts acylation (Figure 18). The chiral analysis by liquid chromatography showed that only one enantiomeric form was synthesised, boosting this methodology as an effective approach to obtain isoflavanones with a high degree of enantiomeric purity [87].

### 2.4. Organocatalysis

**Figure 19:**
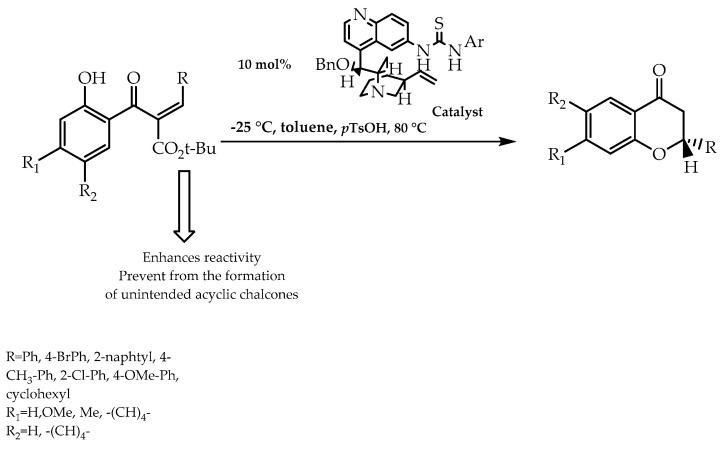
Enantioselective flavanones synthesis via chiral quinine-thiourea catalysis.

Flavonoids can also be obtained through organocatalytic asymmetric processes with the aim of acquiring enantiomeric pure forms of these natural compounds. Biddle et al. [88] proposed an asymmetric synthesis of flavanones based on the intramolecular conjugated addition of α-substituted chalcones, using thiourea compounds as catalysts (Figure 19). The application of this methodology culminated in the synthesis of the flavanone scaffold with 94% of enantiomeric excess [88].

**Figure 20:**
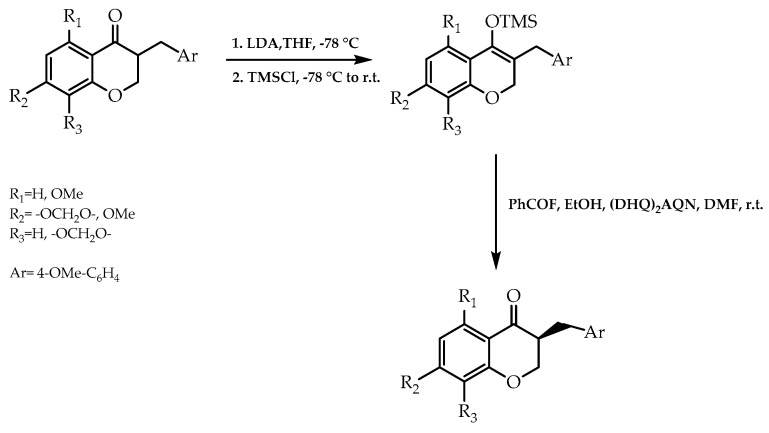
Deracemization of homoisoflavones with silyl enolate formation and subsequent enantioselective protonation.

In 2010, a research team elaborated a deracemization methodology catalysed by alkaloid derivatives to obtain α-substituted ketones [89]. This process encompassed hydrogen fluoride as a proton supplier for the formation of the ammonium cation stemming from the alkaloids’ derivatives. This one, in turn, was responsible for the protonation of the silyl enolate intermediate previously synthesised, giving the desired products. Furthermore, it was proposed that the anion generated as a consequence of the protonation of the amine promoted the catalytic process, therefore enhancing the enantioselective transformation. In order to demonstrate the postulate, and bearing in mind the extent of the biological properties of flavonoids, they employed this strategy in the deracemization of homoisoflavones, resulting in the respective enantiomers with 78% and 81% of enantiomeric excess, and turning this process into a viable route to obtain enantiomeric pure forms of this flavonoid group [89] (Figure 20).

### 2.5. Organometallic Catalysis

**Figure 21:**
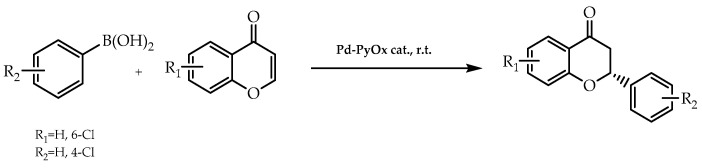
Enantioselective synthesis of flavanones by Pd-PyOx catalysis.

In addition to the enantiomeric pure flavonoids mediated by organocatalysis, organometallic compounds were also employed to promote the stereoselective synthesis of this natural compound. Due to their major interest in obtaining these compounds in the enantiomerically pure form, Lestini et al. [90] focused on the conjugate addition of chromones and arylboronic acids via palladium(II)-pyridinooxazoline catalysis to achieve their goal. For the purpose of enhancing the efficiency of the methodology, they undertook the catalytic process in palladium-nanoreactors with the aim of resulting in a catalytic stability increment. Then, they functionalised pyridinooxazoline with an acrylate monomer, which, in turn, was linked to palladium(II) trifluoroacetate and, subsequently, integrated in the nanoparticle, where the enantioselective synthesis of flavanones occurred. The final products were obtained within a range of from 79% to 84% of enantiomeric excess, highlighting the scientific relevance of this method regarding the enantioselective synthesis of natural bioactive compounds with antitumor, anti-inflammatory, and antimicrobial activities [90] (Figure 21).

**Figure 22:**
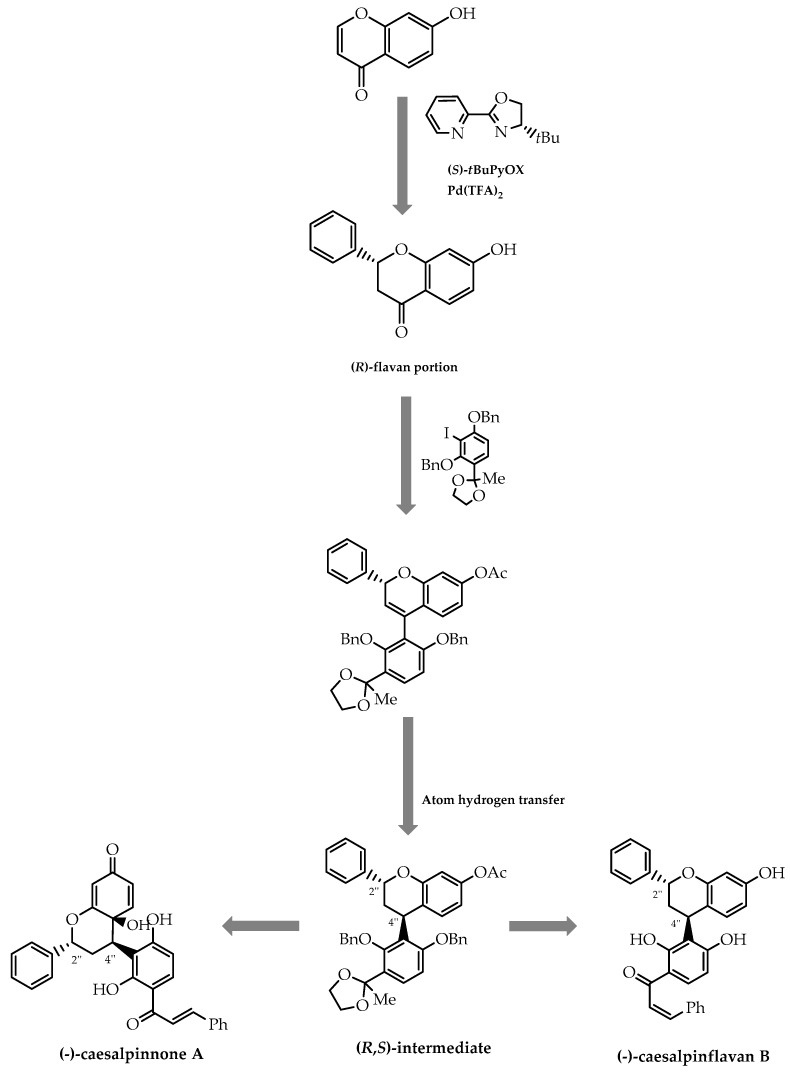
Schematic representation of the stereoselective synthesis of (−)-caesalpinnone A and (−)-caesalpinflavan B via conjugate addition catalysed by Pd-PyOx and atom transfer hydrogen.

Furthermore, a similar process previously developed by Stoltz et al. [91] was employed by Timmerman et al. [92] in the stereoselective synthesis of (-)-caesalpinnone A and (-)-caesalpinflavan: two natural flavonoids with cytotoxic activity against several cancer cell lines [92]. Aiming to accomplish the aforementioned, they proceeded to use the palladium-catalysed conjugation addition methodology to create the sterogenic centre in the flavan portion of caesalpinnone A and caesalpinflavan B [92] (Figure 22). Subsequently, they established the chirality of C4″ in light of the work developed by Shenvi et al. [93], using a hydrogen atom transfer method to reduce the C3″-C4″ bond (Figure 22), resulting in the synthesis of the chiral intermediates of flavan-chalcone hybrids with high enantiomeric excess [92].

**Figure 23:**
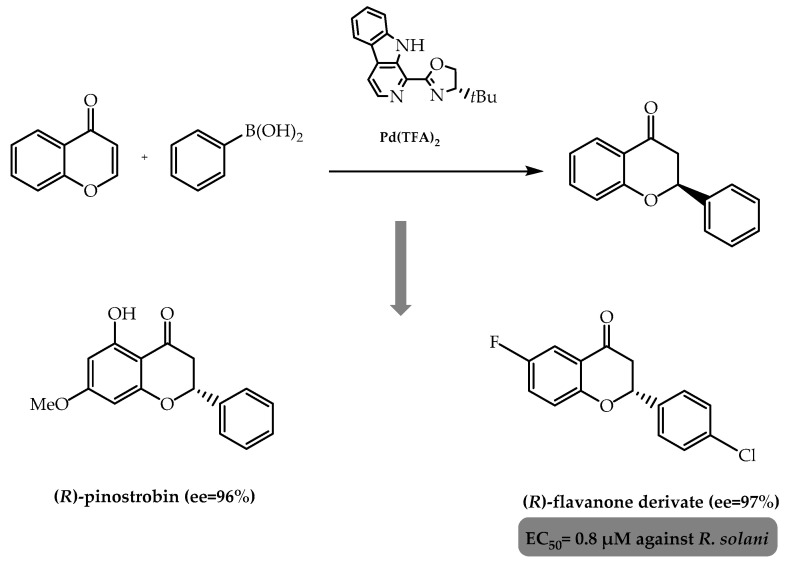
Enantioselective synthesis of flavanones through conjugate addition between chromones and arylboronic acids using Pd-CarOx complex as catalyst.

Moreover, in 2021, Yang et al. [94] focused on improving the palladium catalytic system used in the conjugate addition of arylboronic acids and chromones mentioned above with the goal of obtaining new chiral agrochemicals based on the flavanone scaffold. They successfully unravelled a synthetic route using a palladium-carboline (Pd-CarOx) (Figure 23) to obtain a library of chiral flavanones, in which some of them were synthesised with an enantiomeric excess of 84% to 97%. Subsequently, they established a structure–activity relationship pattern, culminating in the synthesis of (*R*)-pinostrobin through a mild reaction pathway as well as the attainment of the enantiomer *R* of a novel antifungal flavanone-derivative as a promising lead compound [94] (Figure 23).

**Figure 24:**
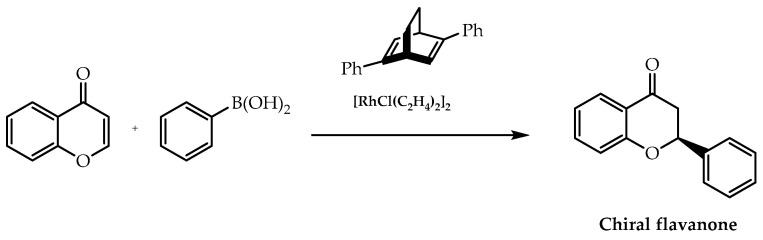
Asymmetric 1,4-addition of arylboronic acids catalysed by rhodium-chiral diene complex.

Additionally, this enantioselective reaction can also be employed using rhodium catalyst complexes. He et al. [95] applied this metallic element with a chiral diene to catalyse the enantioselective synthesis of flavanones via the 1,4-addition of arylboronic acids (Figure 24), resulting in products obtained with enantiomeric excess higher than 97% [95].

### 2.6. Biocatalysis

**Figure 25:**
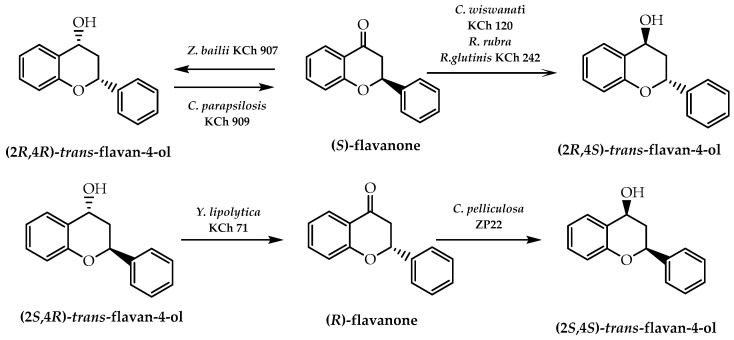
Enantioselective synthesis of flavanones and *cis*/*trans*-flavan-4-ols through biocatalysis using yeast strains.

Biocatalysis presents as a resourceful tool to obtain compounds with structural complexity and several stereogenic centres. In contrast to the conventional chemical synthetic pathway, it can be performed under non-hazardous conditions and foremost enantiomeric excess [96], which makes this type of catalysis an appealing tool for the stereoselective synthesis of natural compounds, including flavonoids. In 2014, Janeczko et al. [97] synthesised chiral flavanones and *cis*/*trans*-flavan-4-ols, which were subject to different yeast strains. This methodology enabled the obtaining of the (2*R*,4*S*)-*trans*-flavan-4-ol from the reduction of (*S*)-flavanone by *C. wiswanati* KCh 120, *R. rubra*, and *R. glutinis* KCh 242 with 92%, 99%, and 98% of enantiomeric excess, respectively [97] (Figure 25). From the reduction of the same chiral flavanone, they were also able to produce (2*R*,4*R*)-*cis*-flavan-4-ol with an enantiomeric excess of 61%, using *Z. bailii* KCh 907, and (2*S*,4*S*)-*cis*-flavan-4-ol was obtained through an (*R*)-flavanone reduction by *C.pelliculosa* ZP22 with an enantiomeric excess of 75% [97] (Figure 25). On the other hand, (*S*)-flavanone and (*R*)-flavanone were obtained through the oxidation of (2*R*,4*R*)-*cis*-flavan-4-ol and (2*S*,4*R*)-*trans*-flavan-4-ol by *C.parapsilosis* KCh 909 and *Y. lipolytica* KCh 71 with enantiomeric excesses of 93% and 85%, respectively [97] (Figure 25).

**Figure 26:**
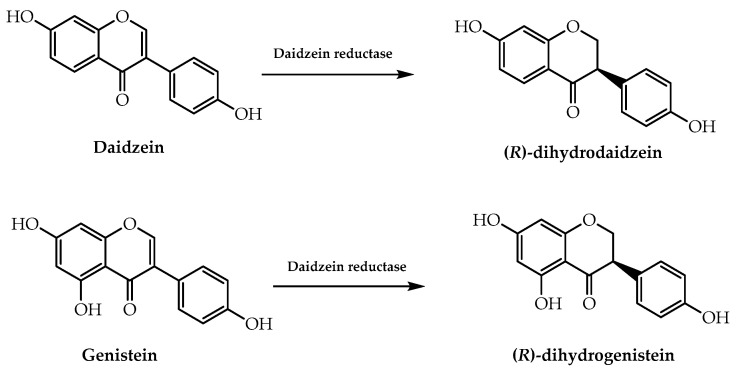
Enantioselective synthesis of (*R*)-dihydrodaidzein and (*R*)-dihydrogenistein by daidzein reductase from *Eggerthella* sp. YY7918.

In light of the therapeutic effect of isoflavones and their derivatives in menopausal disorders and estrogenic-related osteoporosis, Kawada et al. [98] proceeded to evaluate the enzymatic parameters of daidzein reductase, which is intervenient in the conversion of daidzein in the human intestine. According to their results related to enantioselectivity, a highly purified form of the enzyme from *Eggerthella* sp. YY7918 was able to synthesise (*R*)-dihydrodaidzein (Figure 26), disclosing a methodology to obtain enantiomeric pure forms of (*R*)-dihydroisoflavones [98]. Furthermore, they applied this process to another substrate, genistein, enabling them to produce the corresponding (*R*)-dihydroisoflavone [98] (Figure 26).

**Figure 27:**
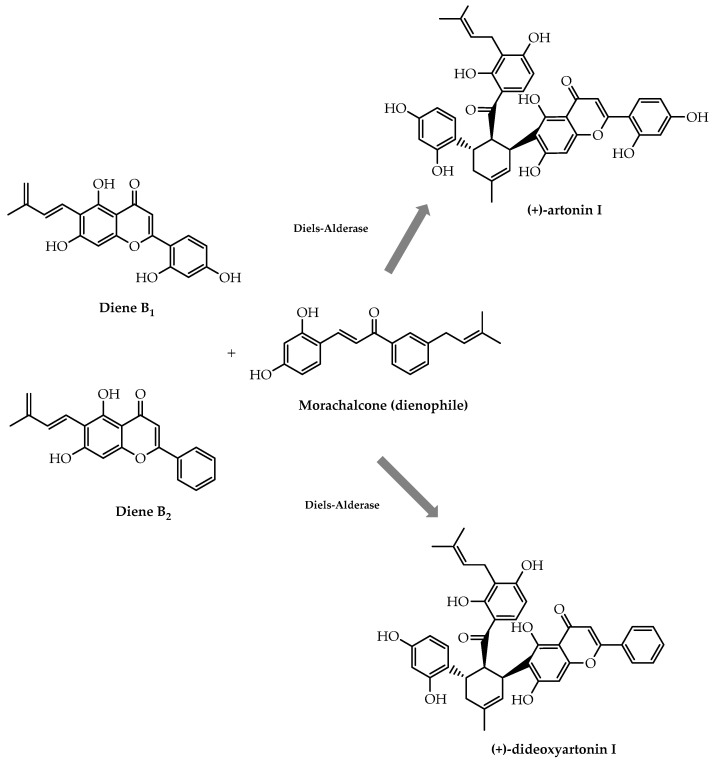
Chemoenzymatic stereoselective synthesis of (+)-artonin I and (+)-dideoxyartonin I.

Another example of the application of biocatalysis to produce enantiomeric pure forms of flavonoids was the employment of a Diels–Alderase to synthesise artonin I, a natural flavonoid with positive effects on *Staphylococcus aureus* multidrug-resistant strains [99]. This enzyme was responsible for the catalysis of the Diels–Alder reaction between morachalcone and the dienes B_1_/B_2_ to give (+)-artonin I and (+)-dideoxyartonin I (Figure 27) with an enantiomeric excess of 99% and higher than 99%, respectively [99].

**Figure 28:**
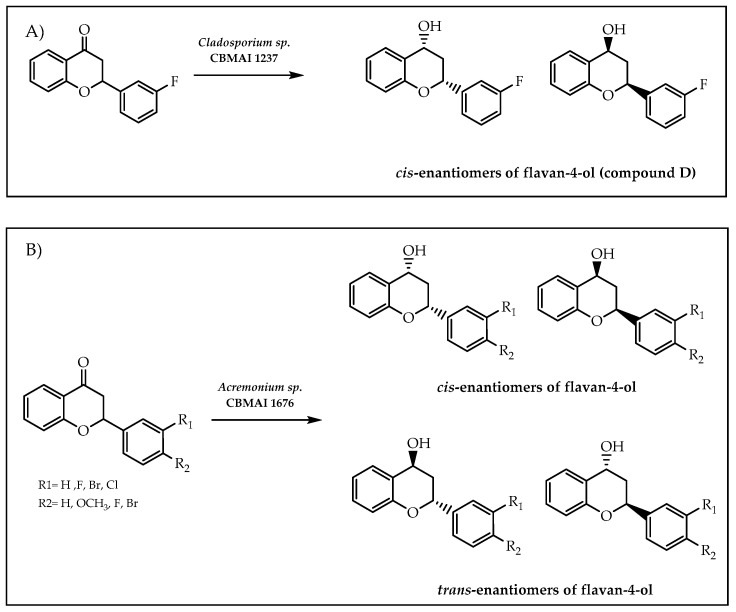
Synthesis of *cis*/*trans*-enantiomers of flavan-4-ols using marine-derived fungi. (**A**) Compound D synthesis by *Cladosporium* sp. CBMAI 1237; (**B**) *Cis* and *trans*-enantiomers of flavan-4-ol synthesis by *Acremonium* sp. CBMAI 1676.

In 2021, de Matos et al. [100] reported the utilisation of strains of marine-derived fungi in order to proceed to the stereoselective reduction of flavanones, culminating in the formation of chiral flavan-4-ols (Figure 28). Pursuant to preliminary results, *Acremonium* sp. CBMAI 1676 and *Cladosporium* sp. CBMAI 1237 were the strains which demonstrated promising results in terms of yield and enantioselectivity and, subsequently, were employed in further studies [100]. From the application of the aforementioned strains, the formation of the *cis*-enantiomers of flavan-4-ol (compound D, scheme A, Figure 28) with an enantiomeric excess of 64% from the activity of *Cladosporium* sp. CBMAI 1237 was highlighted [100]. Additionally, it is also relevant to denote that the synthesis of *cis* and *trans*-enantiomers of the products formed from all flavanones occurred with an enantiomeric excess in a range of 77% to 97% and superior to 95%, respectively, in *Acremonium* sp. CBMAI 1676 [100] (scheme B, Figure 28). As a result, the methodology developed by de Matos et al. [100] enabled the synthesising of halogenated flavanols, particularly brominated flavan-4-ols [100].

**Figure 29:**
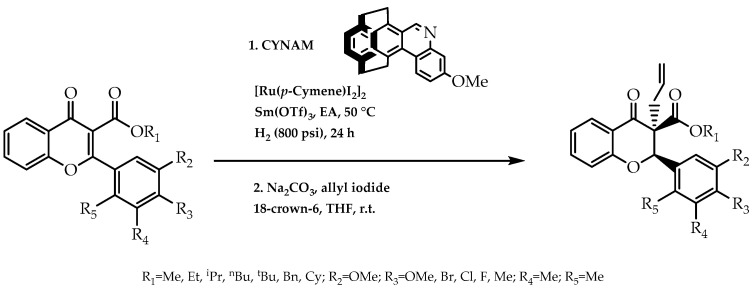
Biomimetic synthesis of chiral flavanones mediated by CYNAM model.

As a consequence of the medicinal relevance of chiral flavanones, Zhu et al. [101] were inspired by a biomimetic asymmetric reduction in NAD(P)H-dependent to synthesise the enantiomeric forms of these flavonoids. They proceeded to elaborate on the chiral [2.2]paracyclophane-based NAD(P)H models (CYNAMs), in which, after reaction conditions’ optimisation, one of the models was applied to obtain enantiomeric tetrasubstituted alkene flavanones (Figure 29), culminating in the formation of most chiral forms in an enantiomeric excess array between 90% and 99% [101]. With this methodology, they were able to reinforce the importance of biocatalysis and the respective cofactors to enable the stereoselective synthesis of flavonoids with higher enantiomeric purity.

### 2.7. Chiral Pool Methodology

**Figure 30:**
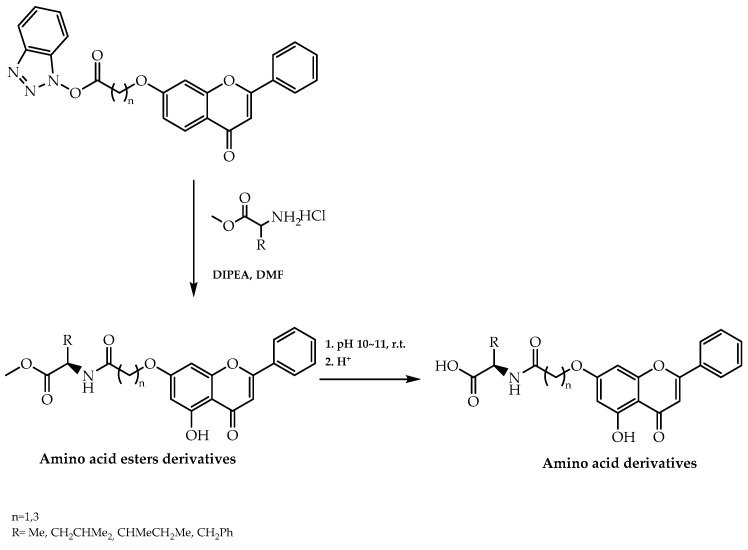
Synthesis of amino acid and amino acid esters derivatives of chrysin by chiral pool approach.

Another approach to achieve highly enantiomerically pure forms of chiral derivatives of flavonoids is through the chiral pool strategy. This method was employed with the aim of synthesising flavonoids with antitumor activity [66]. Chrysin is a natural flavone well-known for its chemopreventive and apoptosis inducer role in several cancer malign forms [102]. Based on the therapeutic relevance of this flavonoid as well as the increasing effect of amino acids in selectivity, Song et al. [102] proceeded to introduce alanine, leucine, isoleucine, and phenylalanine to synthesise the corresponding chrysin amino acid derivatives (Figure 30). As a result of that, an enhancement in the anticancer effect displayed by the obtained products was verified [102], highlighting the importance of chirality in the therapeutic effect of this flavonoid. Moreover, it was also reported that *N*-[4-(5-hydroxy-4-oxo-2-phenyl-4*H*-chromen-7-yloxy)butyryl]-_L_-isoleucine methyl ester demonstrated the most potent inhibitory effect on human gastric carcinoma MGC-803 cells among the synthetic-obtained derivatives and positive control cisplatin, with an IC_50_ value of 3.78 µmol/L [102].

**Figure 31:**
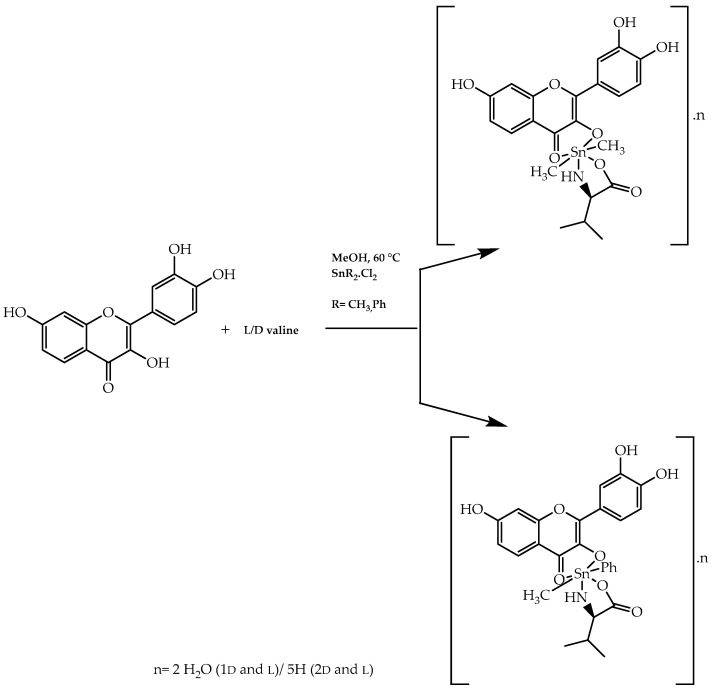
Synthesis of L/D-valine-quercetin diorganotin (IV).

Another illustration of the employment of amino acids in obtaining the flavonoid-related compounds with anticancer activity is the methodology developed by Parveen et al. [103]. They synthesised chiral complexes composed of quercetin, L/D-valine, and organotin (IV), aiming to achieve a synergetic effect from these three components (Figure 31). From further cytotoxic studies carried out in HeLa (cervix), MCF7 (breast cancer), Hep-G2 (liver cancer), and MIA-Pa-Ca-2 (pancreatic cancer), it was possible to verify that the majority of the L-enantiomers of the complexes showed values of GI_50_ lower than 10 µg/mL, outlining their potential in chemotherapy [103]. Additionally, molecular docking studies revealed that the configuration was a preponderant factor in the interaction between the target and L-valine-quercetin diorganotin (IV) complexes and, as a consequence, it corroborated the role of chirality on the pharmacological effect demonstrated by these synthesised compounds [103].

**Figure 32:**
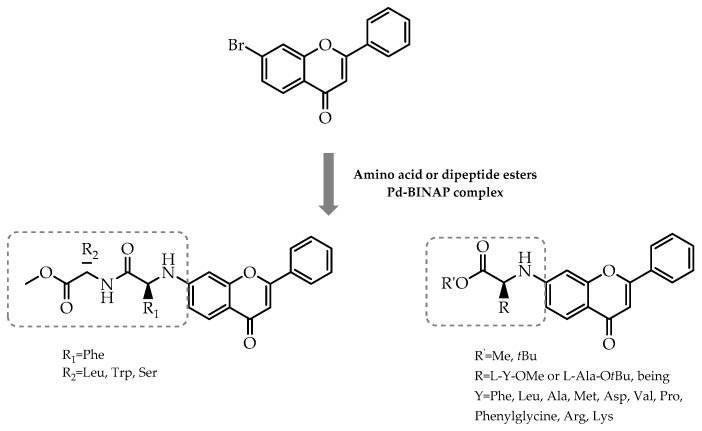
Schematic representation of flavone-amino acid hybrids synthesis via Buchwald–Hartwig reaction.

Moreover, the work of Pajtás et al. [104] constituted another contribution to the employment of amino acids and peptide moieties in flavonoids. As reported by them, the insertion of these chiral molecules via the Buchwald–Hartwig amination of bromoflavones in the presence of BINAP and palladium as a catalyst complex averted the racemisation of the resulting products, culminating in the enantiomeric pure forms of flavone derivatives [104] (Figure 32). Furthermore, these compounds were, subsequently, tested in vitro for cytotoxic activity, in which a compound revealed significant cytotoxic activity (95.43% in a concentration of 50 µM) in the U87 glioblastoma cell line [104].

**Figure 33:**
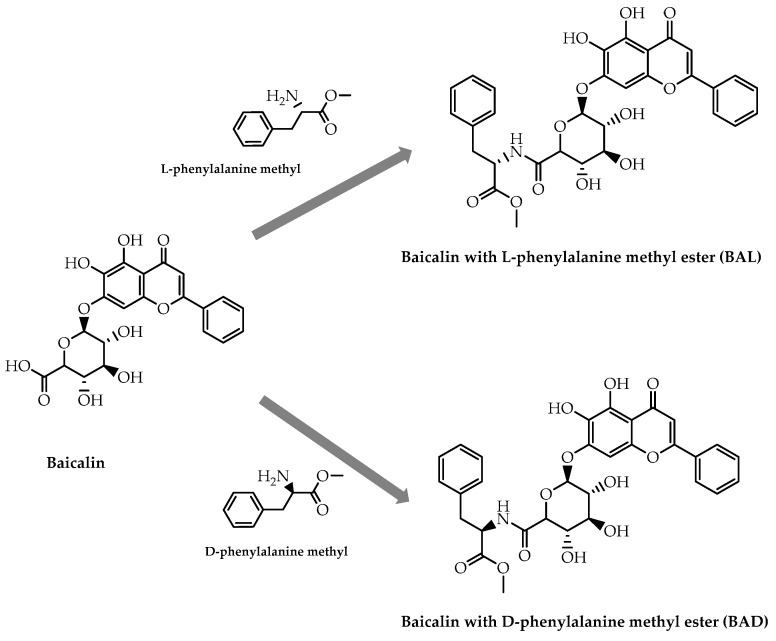
Schematic representation of the synthesis of baicalin phenylalanine methyl esters derivatives.

More recently, Hou et al. [105] synthesised enantiomeric forms of baicalin derivatives, combining this natural flavonoid with phenylalanine methyl esters in order to improve antitumor activity (Figure 33). As predicted, the introduction of this chiral amino acid ester increased the inhibitory effect on cancer cell growth, particularly in A549 cells, exhibiting an inhibition rate of 88.95% at 48 h in a concentration of 50 µg/mol for baicalin with L-phenylalanine methyl ester (BAD), and an inhibition rate of 94.13% for baicalin with D-phenylalanine methyl ester (BAL) [105]. Furthermore, immunohistochemistry data showed that these baicalin derivatives suppressed tumor angiogenesis, with BAL being more potent than BAD [105]. These results confirm that the molecular modification of flavonoids with different enantiomeric forms of natural chiral molecules, such as amino acids, could result in bioactive compounds with different potency.

**Figure 34:**
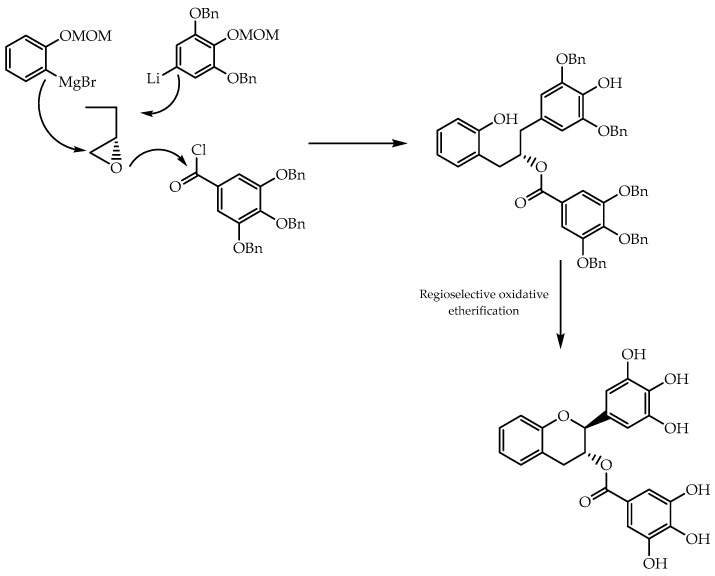
Production of *trans*-flavan-3-ols gallates from epichlorohydrin using chiral pool approach.

The chiral pool method was also used for other building blocks, namely epichlorohydrin. Shiraishi et al. [106] synthesised enantiomeric forms of *trans*-flavan-3-ol gallates, using (*S*) and (*R*)-epichlorohydrine (Figure 34) as an integrant part of 1,3-diaryl-2-propanols, which are intermediates in this reaction pathway. The final products were, subsequently, obtained by regioselective oxidation etherification with 2,3-dichloro-5,6-dicyano-1,4-benzoquinone, and were screened after for anticancer activity [106]. From the experiments in the U266 cell line (multiple myeloma), it was possible to observe that both enantiomers displayed similar IC_50_ values, suggesting that chirality might not be a detrimental feature for the antitumor effect of the obtained *trans*-flavan-3-ol gallates [106].

### 2.8. Other Synthetic Methologies

**Figure 35:**
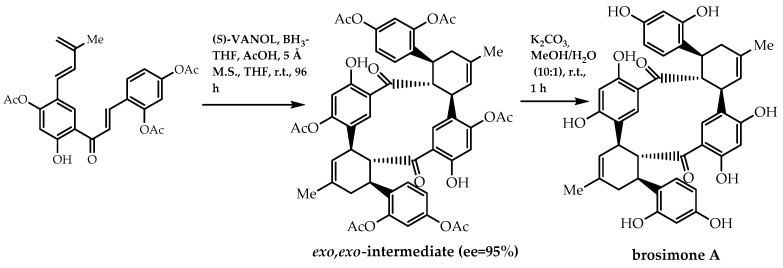
Synthesis of (-)-brosimone A.

**Figure 36:**
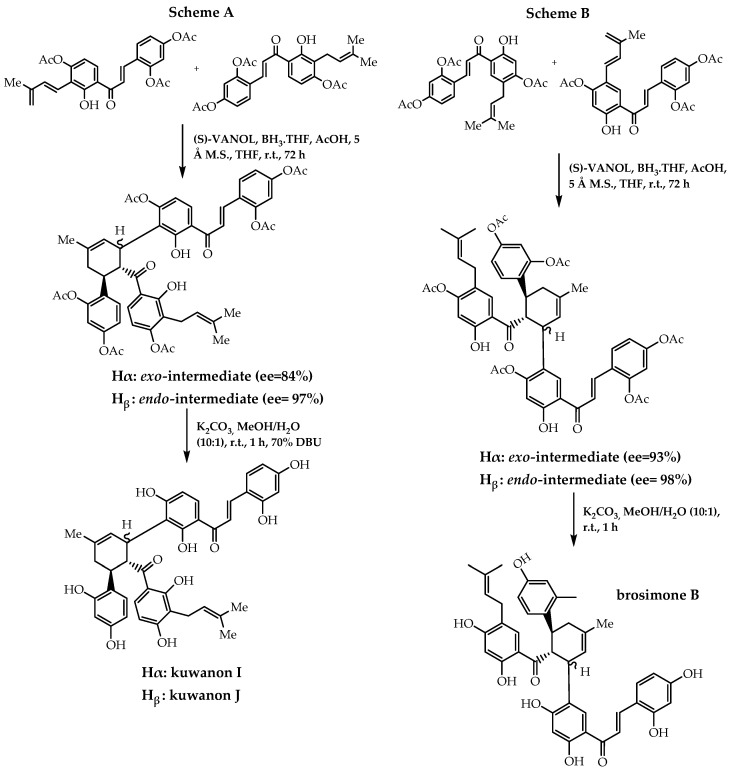
Synthesis of (−)-kuwanon I, (+)-kuwanon J, and (−)-brosimone B. **Scheme A:** Stereoselective biomimetic total synthesis of (−)-kuwanon I and (+)-kuwanon J; **Scheme B:** Stereoselective biomimetic total synthesis of (−)-brosimone B.

There are also reports of other synthetic processes with the goal of obtaining enantiomeric pure forms of flavonoids, namely the Diels–Alder reaction. As promising anticancer, anti-inflammatory, and antiviral agents, prenylflavonoids have been arousing interest from researchers. In 2014, Han et al. [107] reported a stereoselective biomimetic total synthesis of (-)-brosimone A (Figure 35), (-)-kuwanon I (scheme A, Figure 36), (+)-kuwanon J (scheme A, Figure 36), and (-)-brosimone B (scheme B, Figure 36). In order to establish the stereogenic centres of these Diels–Alder natural products, they resorted to an asymmetric Diels–Alder cycloaddition of a 2′-hydroxychalcone derivative, using a chiral boron-VANOL complex as the catalyst. Lately, they have employed this methodology to obtain chalconoids (-)-nicolaioidesin C and (-)-panduratine A, with 96% and 87% of enantiomeric excess, respectively [108].

**Figure 37:**
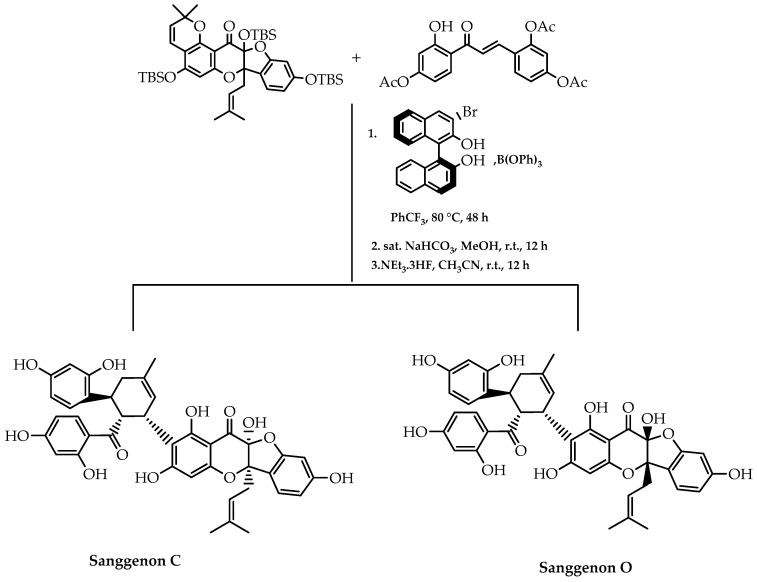
Stereoselective synthesis of sanggenon C and O via [4+2] cycloaddition based on stereodivergent RRM.

Another method to synthesise Diels–Alder natural products was demonstrated by Qi et al. [109]. They embarked on a strategic stereodivergent reaction of a racemic mixture (RRM) to obtain (+)-sanggenon C and (-)-sanggenon O, involving an asymmetric [4+2] cycloaddition catalysed by a boron-BINOL complex (Figure 37). Using this reaction process, these flavonoid derivatives were obtained with an enantiomeric excess of 98% and 93%, respectively [109].

**Figure 38:**
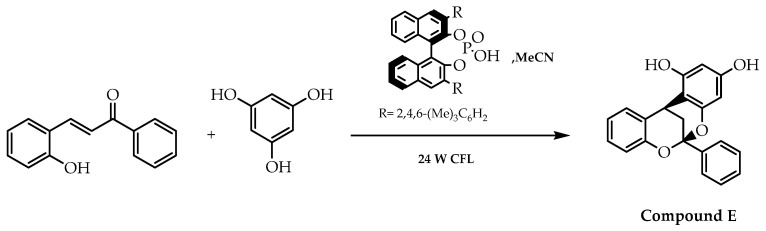
Bio-inspired stereoselective synthesis of phloroglucinol–flavonoid hybrid.

As prior demonstrated, enantioselective biomimetic reactions enable synthesising chiral flavonoids with diverse biological activities. Taking into consideration the anticancer, anti-inflammatory, antioxidant, and antibacterial potential of hybrid flavonoids, Gao et al. [110] developed a methodology based on the asymmetric coupling of 2-hydroxychalcone using an appropriate Brønsted acid as the catalyst, an adequate nucleophile, and a visible light as the reaction promotor [110]. Subsequently, this photochemical bio-inspired reaction was applied to obtain enantiomeric forms of hybrid flavonoids with indole, cyclohexa-1,3-dione, or phloroglucinol, highlighting the formation of the 2-hydroxychalcone phloroglucinol hybrid (compound E, Figure 38) as a result of the counter-anion-directed enantioselective addition of 2-hydroxychalcone and phloroglucinol with an enantiomeric excess of 70% [110].

**Figure 39:**
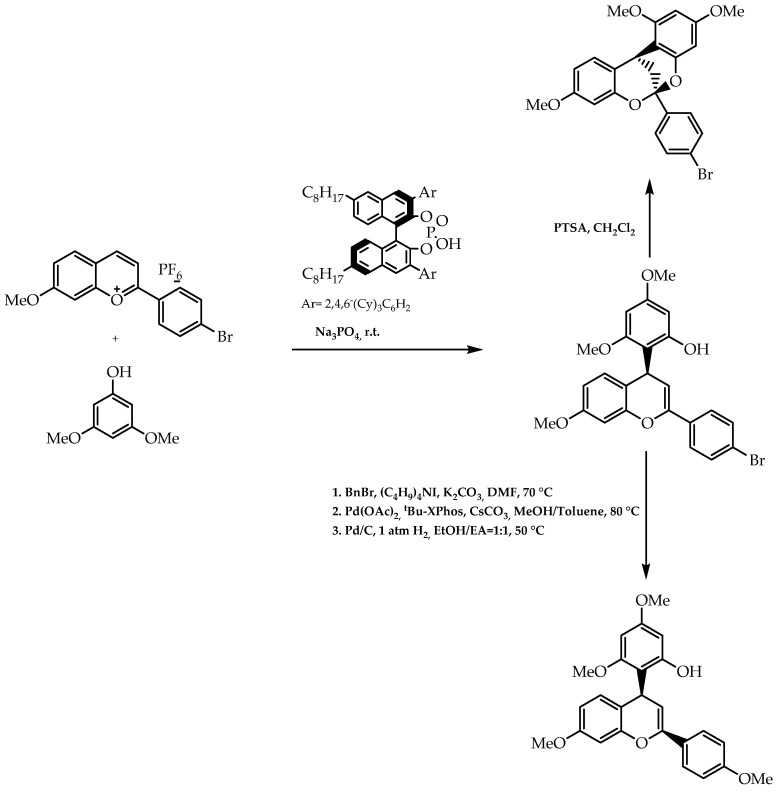
Stereoselective synthesis of scaffolds integrated in flavonoid-related compounds by chiral anion phase-transfer.

Another example of the implementation of a biomimetic reactional approach is the synthetic methodology developed by Yang et al. [111]. This approach was based on the application of a chiral anion phase in order to promote the addition of nucleophilic phenols to benzopyrylium salts (Figure 39), synthesising 2,4-diarylbenzopyran and 2,8-dioxabicyclo [3.3.1]nonane with enantiomeric excesses of 91% and 94%, respectively [111]. These scaffolds have crucial importance from a synthetic point of view due to the fact that they integrate flavonoid-related compounds [111]; therefore, a reaction pathway was unfolded to access natural products with a diversified array of biological activities.

**Figure 40:**
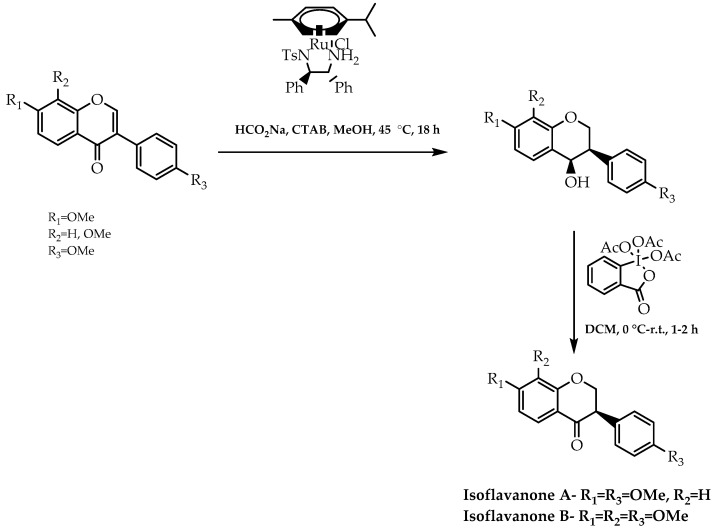
Enantioselective synthesis of isoflavanones via asymmetric ATH-DKR and DMP oxidation.

More recently, in this thematic field of stereoselective synthesis, and inspired by Metz et al. [112,113] and their previous works [114], Gaspar et al. [115] were able to enlarge the scope of ATH-DKR to obtain *cis*-3-phenylchroman-4-ols and, subsequently, use them as intermediates for the synthesis of chiral isoflavanones, which possess crucial biological activities [116]. With the aim of accomplishing their goal, they applied a Noyori–Ikariya ruthenium complex as the catalyst and sodium formate as the hydrogen source to the reaction (first reaction step, Figure 40), culminating in the formation of (*R*,*R*)-*cis*-alcohols in a range between 92% and 99% of the enantiomeric ratio [115]. Thereafter, they used a Dess–Martin periodinane (DMP) oxidation to synthesise two chiral natural isoflavanones (second reaction step, Figure 40), maintaining the enantiomeric ratios previously acquired [115].

## 3. Conclusions

Flavonoids are natural polyphenolic compounds mainly found in plants and associated with a wide range of biological activities, including antiviral, antimicrobial, antitumor, and antioxidant activities. They can be also employed in the cosmetic, food, textile, and metallurgic fields.

Owing to their biological relevance, flavonoids have been arousing interest and, as a result, synthetic methodologies have been employed in order to obtain these natural compounds, namely the following: Algar–Flynn–Oyamada, Allan–Robinson, Baker–Venkataraman, Claisen–Schmidt, Karl von Auwers, Kostanecki, Mentzer Pyrone, Suzuki–Miyaura, deoxybenzoin route, reductive cleavage of isoxazoles, intramolecular ketene cycloaddition followed by decarboxylation, rearrangement and cyclisation of chalcone epoxides, rearrangement of flavanones, Wacker–Cook tandem conversion of α-methylene deoxybenzoins, Cu(I)-mediated cyclisation of 3-(2-bromophenyl)-3-oxopropanol, Pechmann reaction, Perkin reaction, Wittig reaction of benzophenones, metal-catalysed cross-coupling reactions, and direct arylation of arylboronic acids to coumarins through palladium-catalysed oxidative Heck coupling.

Regarding stereoselective synthesis, many strategies were explored such as chalcone epoxidation, Sharpless asymmetric dihydroxylation, the Mitsunobu reaction, and the cycloaddition of 1,4-benzoquinones with 2*H*-chromenes via Ti-TADDOLate catalysis. Chiral auxiliaries were also applied in the synthesis of flavonoids enantiomers, highlighting imidazolidinone in the α-benzylation reaction of phenyl acetic acid derivatives and (*S*,*S*)-(+)-pseudoephedrine in an asymmetric aldol reaction. Moreover, organocatalytic processes were used with the aim of attaining enantiomeric pure forms of these natural compounds, enhancing the employment of thiourea and alkaloid moieties in the intramolecular conjugate addition of α-substituted chalcones and deracemization of homoisoflavones, respectively. Furthermore, organometallic complexes were also used with the aim of synthesising chiral flavonoids, namely palladium-pyridinooxazoline/carboline and rhodium in the reaction of the addition of chromones to arylboronic acids. Biocatalysis is an environmentally sustainable tool to proceed with the synthesis of enantiomeric forms of these polyphenolic compounds, highlighting the production of chiral flavanones and *cis*/*trans*-flavan-4-ols by yeast strains, (*R*)-dihydroisoflavone synthesis by daidzein reductase from *Eggerthella* sp. YY7918, Diels–Alderase application, the stereoselective reduction in flavanones by marine-derived fungi to obtain chiral flavan-4-ols, and the development of chiral NAD(P)H models such as CYNAMs. The chiral pool was also reported as a synthetic route to acquire flavonoid derivatives, mainly by the employment of amino acids and epichlorohydrin. Although the employment of the methodologies mentioned above enabled the obtaining of the enantiomeric pure forms of flavonoids with high enantiomeric excess, the development of novel approaches in order to encompass the synthesis of other flavonoids classes is still required. Henceforward, the study of the biological properties of the chiral flavonoids obtained by the methods mentioned above is of crucial importance from a scientific perspective, and to further explore their pharmacological potential as well as to perform enantioselectivity studies.

## Data Availability

Not applicable.
